# Fat Utilization During High-Intensity Exercise: When Does It End?

**DOI:** 10.1186/s40798-016-0060-1

**Published:** 2016-08-31

**Authors:** Ratko Peric, Marco Meucci, Zoran Nikolovski

**Affiliations:** 1 Institute for Sport and Occupational Medicine Banja Luka, Zdrave Korde 4, 78000 Banja Luka, Bosnia and Herzegovina; 2 Department of Health and Exercise Science, Appalachian State University, Boone, NC USA; 3 Department of Biochemistry, Aspire Academy, Doha, Qatar

**Keywords:** Fat, Oxidation, High intensity, Anaerobic threshold, Running

## Abstract

**Background:**

This study examined substrate oxidation at high-intensity exercise and aimed to determine when fat oxidation ends (FAT_min_). We hypothesized the existence of a connection between the anaerobic threshold (AnT) and FAT_min_ point.

**Methods:**

Breath-by-breath data obtained from indirect calorimetry during a graded treadmill test were used to measure substrate oxidation and maximal oxygen uptake (VO_2max_) on 47 males (30 athletes (ATL) and 17 non-athletes (NATL)). Pearson correlation coefficient (*r*) and effect size (*R*^2^) were used to test correlations between VO_2_ at AnT and at FAT_min_.

**Results:**

Maximal oxygen uptake (VO_2max_) was 56.17 ± 4.95 and 46.04 ± 3.25 ml kg^−1^ min^−1^ in ATL and NATL, respectively. In ATL, AnT was observed at 87.57 ± 1.30 % of VO_2max_ and FAT_min_ was observed at 87.60 ± 1.60 % of VO_2max._ In NATL, AnT and FAT_min_ were at 84.64 ± 1.10 % of VO_2max_ and 85.25 ± 1.10 % of VO_2max_, respectively. Our data show large correlations between VO_2_ at AnT and VO_2_ at FAT_min_ for ATL (*r* = 0.99, *p* < 0.01, 95 % CI 0.99 to 1.00) and NATL (*r* = 0.97, *p* < 0.01, 95 % CI 0.91 to 0.98). The effect size of correlations for ATL and NATL were 0.98 and 0.94, respectively.

**Conclusions:**

Our results show high correlation between AnT and FAT_min_ in both ATL and NATL with equal substrate oxidation rates at AnT.

## Key Points

- A high correlation between FAT_min_ and anaerobic threshold (AnT) exists in both athletes and recreationally active males.
- CHO and fat utilization at AnT are equal in both groups.
- At intensities above AnT, no presence of lipids in energy expenditure was observed.

## Background

It is well documented that carbohydrates (CHO) and lipids are simultaneously oxidized during energy production. Although proteins are also involved in this process, their contribution to energy expenditure is minimal [17]. At the onset of exercise, there is an increase of glycolysis, which drops after several minutes if low or moderate intensity is maintained. During that time, there is an increase in fat oxidation in the muscle due to stimulation of lipolysis in adipose tissue, augmented muscle blood flow, possibly enhanced translocation of FAT/CD36 protein into the cell membrane, and the possibility of stimulated lipolysis in the muscles themselves through increased levels of hormone-sensitive lipase (HSL) [8]. Intensity of the exercise where there is maximal utilization of lipids as a fuel is referred to as FAT_max_, which is correlated with aerobic threshold (AerT) [3, 5, 9]. In contrast to CHO oxidation, which increases together with the work rate, absolute fat oxidation rate declines at high-intensity exercises. Achten et al. [1] introduced the point where fat utilization becomes negligible (FAT_min_).

During prolonged activities at high-intensity exercises, the main energy source is CHO derived from the muscle and liver glycogen [26]. However, since the body can only store a limited amounts of this metabolic substrate (up to 600 g), prolonged high-intensity physical activity above FAT_min_ could lead to depletion of glycogen stores, therefore making FAT_min_ an important parameter to consider when exercising at high-intensity for long periods of time.

Few studies exist which were performed systemically and accurately with the aim of determining the intensity at which FAT_min_ is reached. There are divergent study findings with previous research since some have shown negligible fat oxidation in cyclists and runners at intensities of approximately 80 to 95 % of maximal oxygen uptake (VO_2max_) [1–3, 27]. Yet, others report that fat oxidation was not negligible in cyclist and runners while exercising at the 86 and 95 % of VO_2max_ [13, 28]. The wide range of intensities reported leads to inconsistency of data obtained and may be a consequence of differences between subject fitness levels and a lack of consistency of testing methodologies.

The mechanism behind fat utilization at high intensities has not been fully elucidated and the topic warrants further discussion. Horowitz and Klein [14] support the hypothesis that reduction of fat oxidation during high-intensity exercise is caused by increased trapping of fatty acids within adipose tissue due to a decrease in blood flow and insufficient removal by the bloodstream. Another factor which can limit fat mobilization during high-intensity exercise is the lactate accumulated in the blood. Lactate promotes re-esterification of free fatty acids (FFA) produced during lipolysis limiting the entry of lipids into the bloodstream [19]. Dyck et al. [9] reported when increased fat is available in the plasma at high-intensity exercises (above 80 % of VO_2max_), a reduction of glycolysis is observed and the breakdown of muscle glycogen decreases. Also, it has been shown that allosteric regulators (inorganic phosphate (Pi) and adenosine monophosphate (AMP)) play a major role in the regulation of glycogenolysis [17]. Van Loon et al. [29] reported low fat utilization during high-intensity exercises hypothesizing that carnitine acts as a sink for acetyl group storage during continuous accumulation of muscle acetyl carnitine when oxygen deficit occurs.

The second ventilatory threshold (VT_2_) or AnT is often considered a point of a transition from aerobic to anaerobic metabolism and a well-established marker of an individual’s endurance capacity [12]. It varies from person to person and sport to sport. Due to the high individuality of each parameter, we hypothesize that FAT_min_ could correlate highly with AnT making it a valid indicator of substrate utilization and the end point of fat oxidation.

Therefore, the primary aim of this study was to assess the substrate oxidation at high-intensity physical activity and to determine the point at which FAT_min_ occurs in athletes and non-athletes during a continuous incremental treadmill test. Furthermore, we aimed to explore an existence of relationships between FAT_min_ and AnT.

## Methods

### Participants

Forty-seven healthy, non-smoking males (30 athletes (ATL) and 17 non-athletes (NATL)) participated in this study. ATL (basketball, football, and handball) competed at national or international levels and undertook ≥13 h of training per week over the last 8 years. NATL (recreational football) performed ≤2 h of physical activity per week for the last 6 months. Subjects were recruited through a voluntary, open-access, online poll. Prior to testing, all subjects completed a questionnaire regarding their exercise and health histories. Informed consent was obtained from all individual participants for whom identifying information is included in this article. This research was conducted according to the policies and a guideline provided by the Declaration of Helsinki and was approved by the Institutional ethics committee. Anthropometric values for both groups are presented in Table 1. The body mass and height were assessed as they reported to the laboratory for the first time using a Seca 763 digital medical scale and stadiometer (SECA_®_, Hamburg, Germany). Subjects were asked to abstain from any laborious physical activity 24 h prior to the testing and instructed to have their last meal 2 h before the testing session. Also, they were asked to refrain from consuming caffeine and nutritional supplements on the testing day. Pre-test meals were not standardized. Testing was performed between 09:00 and 12:00 h in the winter period. The laboratory where the study was performed was located 270 m above sea level, and standard conditions were maintained (21 °C and 43 % humidity).

**Table 1:** Anthropometric values of the subjects

	Athletes (*n* = 30)	Non-athletes (*n* = 17)
Age (year)	25.3 ± 3.2	26.8 ± 3.1
Height (cm)	197 ± 8.6	176.5 ± 6.5
Body mass (kg)	99 ± 6.3	85.6 ± 6.3

Values are expressed as means ± SD

*n* number of subjects

### Exercise Protocols

All subjects performed a graded exercise testing (GXT) on a T170D motorized treadmill (COSMED_®_, Rome, Italy) until volitional exhaustion to measure gas concentration and to calculate the oxidation rate. All subjects were experienced in treadmill exercise testing. Oxygen consumption (VO_2_) and carbon dioxide production (VCO_2_) were assessed during exercise using a breathe-by-breath Quark PFT Ergo (COSMED_®_, Rome, Italy) system. Prior to each test session, a two-point gas calibration for oxygen and carbon dioxide (O_2_ 16.10 and 20.93 %; CO_2_ 0.00 and 5.20 %, respectively) and a turbine flow meter calibration (3-L syringe) were performed according to the manufacturers’ recommendation. Fluctuations of breath-by-breath data were minimized using a six-breath smoothing and consequent 30-s averaging. An experienced exercise physiologist determined AerT and AnT manually, using the methods described by Meyer et al. [21]. VO_2max_ was determined using the following criteria: a respiratory quotient (RQ) ≥1.15 or a plateau of VO_2_ in spite of a load increase [15]. The GXT protocol used consisted of three stages: rest, exercise, and recovery. The resting stage of 2 min standing was performed to obtain baseline values. The exercise stage started at 6 km h^−1^ speed and constant 1 % incline, followed by an increase in speed of 1 km h^−1^ every 2 min until volitional exhaustion. A 3-min active recovery was performed immediately after stopping and required the subjects to run for a further 3 min at the starting speed in order to observe physiological recovery of the subjects. To test the reliability of the protocol, a repeat test was performed on ten randomly selected subjects under identical laboratory conditions and daytime to avoid circadian variance. VO_2max_ data obtained were compared with the initial test.

### Metabolic Calculations

Substrate oxidation was estimated continuously during GXT by using modified stoichiometric equation from Elia and Livesey [10], which assumed negligible contribution of protein oxidation.

- RQ = VCO_2_/VO_2_ if calculated RQ < 0.7 than RQ = 0.7 or if calculated RQ > 1.0 than RQ = 1.0.
- CHO % = (5.045 × RQ − 3.582) / (0.36 × RQ + 1.103) if CHO % < 0 than CHO % = 0 or if CHO % > 1 than CHO % = 1.0
- FAT % = 1.0 − CHO %

VO_2max_ was expressed as maximum amount of oxygen in milliliters, used per kilogram of body weight in 1 min (ml kg^−1^ min^−1^). Fat and CHO utilization was expressed in grams per minute (g min^−1^) or as percentage of the total energy production. The test results for each individual were used to create a fat oxidation curve that was in turn used to determine FAT_min_.

### Statistical Analysis

Data were analyzed and presented as mean ± standard deviation (SD), 95 % confidence interval (CI), and lowest to highest values using MedCalc 12 (MEDCALC_®_, Ostend, Belgium) statistical software. Normal distribution of all measured variables was assessed using the Shapiro-Wilk test with normal distribution accepted for all variables. A Student *t* test was used to test-retest reliability of VO_2max_ protocol with no statistical differences observed (*p* = 0.76). To assess differences in measured parameters between ATL and NATL, we used Mann–Whitney *U* test. A Pearson product-moment correlation coefficient (*r*) was used to assess the linear relationship between VO_2_ at AnT and VO_2_ at FAT_min_. Coefficient of determination (*R*^2^) was used to detect the effect size and strength of relationships. Alpha intervals for all hypothesis testing were set at *p* ≤ 0.05 as the level of significance for statistical tests unless stated otherwise.

## Results

**Fig. 1:**
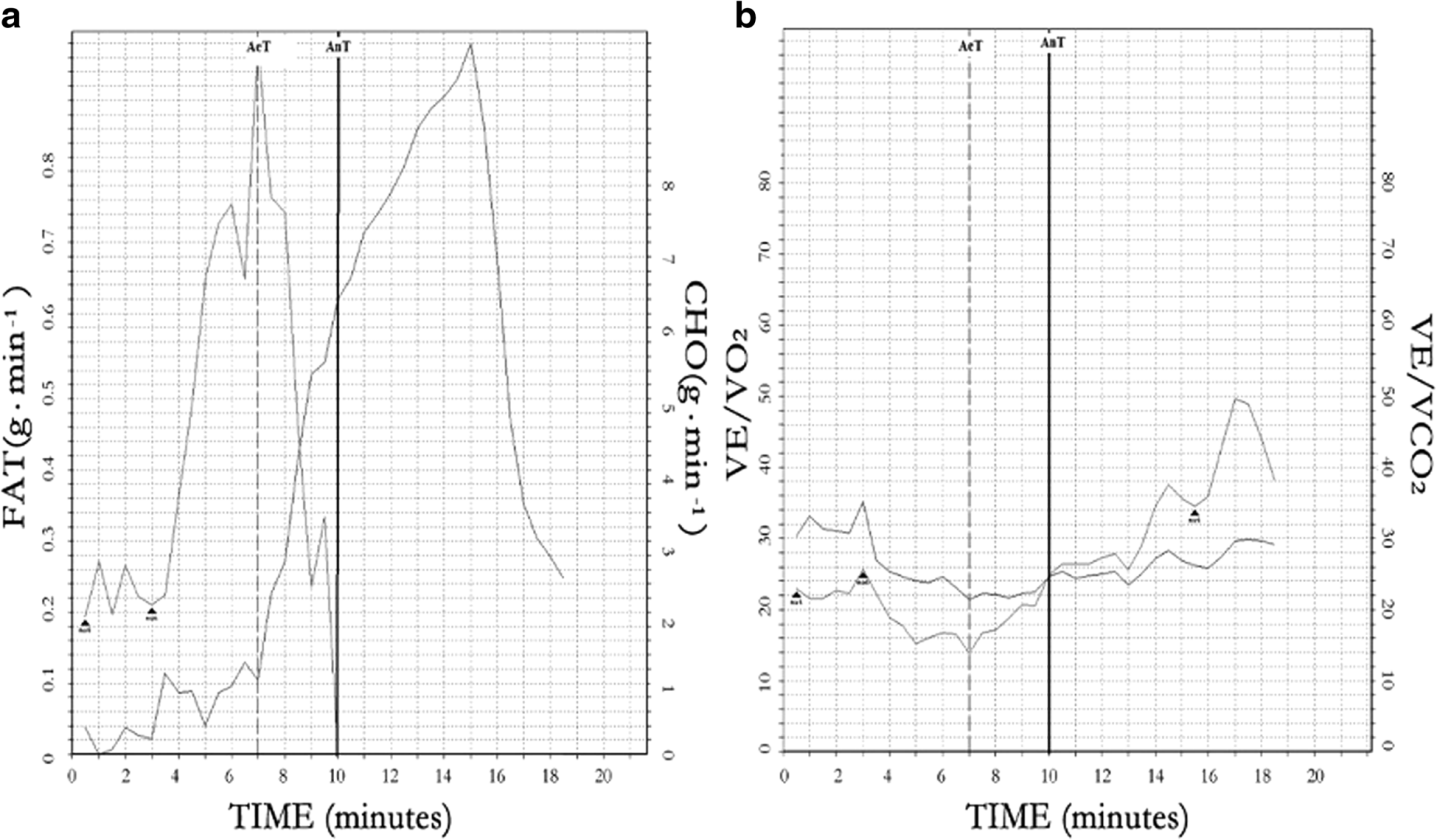
Graphic representation of correlation between AerT and FAT_max_ and AnT and FAT_min_. **a** Fat and CHO utilization (g min^−1^) at AerT and AnT in tested subjects (PFT Suite Software, COSMED_®_, Rome, Italy). **b** Threshold detection method by EqO_2_ and EqCO_2_ principles [21]

The subjects’ metabolic values are presented in Table 2. As expected, higher VO_2max_ results were found in ATL (95 % CI 54.32 to 58.05) compared to NATL (95 % CI 44.37 to 47.71) (*U* = 0.00, *p* < 0.01). AerT was reached at a 37.01 ± 5.33 ml kg^−1^ min^−1^ VO_2_ (95 % CI 34.02 to 39.56) in ATL and at a 23.59 ± 4.51 ml kg^−1^ min^−1^ VO_2_ (95 % CI 21.49 to 25.33) (*U* = 28.00, *p* < 0.01) in NATL. Fat utilization increased with exercise intensity until the AerT was reached, followed by a decrease of total fat oxidation and an increase of CHO utilization (Fig. 1).

**Table 2:** Subjects metabolic data

	Athletes (*n* = 30)	Non-athletes (*n* = 17)
VO_2max_ (ml kg^−1^ min^−1^)	56.17 ± 4.95	46.04 ± 3.25
AerT (% VO_2max_)	65.89 ± 1.80	51.25 ± 2.10
AnT (% VO_2max_)	87.57 ± 1.30	84.64 ± 1.10

Values are expressed as means ± SD

*n* number of subjects, *AerT* aerobic threshold, *AnT* anaerobic threshold, *VO*_*2max*_ maximal oxygen uptake

AnT was reached at a 49.19 ± 6.52 ml kg^−1^ min^−1^ VO_2_ (95 % CI 46.76 to 51.63) in ATL and at a 38.97 ± 3.26 ml kg^−1^ min^−1^ VO_2_ (95 % CI 37.29 to 40.65) in NATL (*U* = 27.00, *p* < 0.01).

FAT_min_ was obtained at 49.22 ± 6.53 ml kg^−1^ min^−1^ of VO_2_ (95 % CI 46.78 to 51.66) or at 87.60 ± 1.60 % of VO_2max_ for ATL and 39.25 ± 3.44 ml kg^−1^ min^−1^ of VO_2_ (95 % CI 37.48 to 41.02) or at 85.25 ± 1.10 % of VO_2max_ for NATL (*U* = 29.00, *p* < 0.01).

**Fig. 2:**
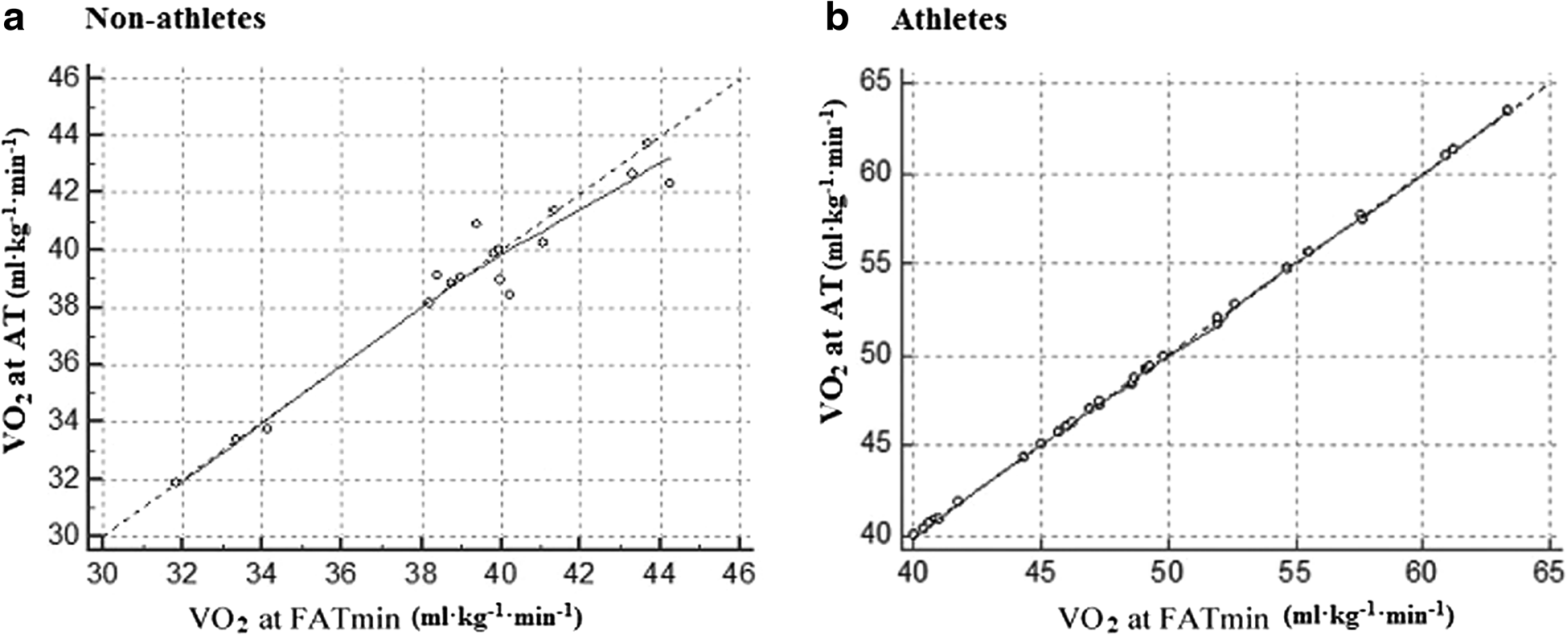
Pearson correlation between VO_2_ (ml kg^−1^ min^−1^) at AnT and at FAT_min_ for NATL (*r* = 0.97, *p* ≤ 0.01, 95 % CI 0.91 to 0.98) (**a**) and ATL (*r* = 0.99, *p* ≤ 0.01, 95 % CI 0.99 to 1.00) (**b**) with line of equality

Pearson correlations between VO_2_ at AnT and at FAT_min_ in ATL (Fig. 2b) and NATL (Fig. 2a) was high (*r* = 0.99, *p* < 0.01, 95 % CI 0.99 to 1.00 and *r* = 0.97, *p* < 0.01, 95 % CI 0.91 to 0.98), respectively. Effect size for ATL was *R*^2^ = 0.98 explaining for 98.01 % of variance. NATL had *R*^2^ = 0.94 effect size explaining 94.09 % of variance.

Fat oxidation at AnT in ATL and NATL was 0.00 ± 0.00 g min^−1^ (95 % CI −0.00 to 0.00) and 0.02 ± 0.02 g min^−1^ (95 % CI 0.00 to 0.03) (*U* = 168.00, *p* < 0.01), respectively.

CHO oxidation at AnT in ATL averaged 4.47 ± 1.24 g min^−1^ (95 % CI 4.01 to 4.93) and provided 97.91 ± 1.02 % (95 % CI 97.52 to 98.29) of the total energy expenditure. The lowest value was 94.50 % and the highest 99.20 %. CHO utilization in NATL at AnT was equal to 4.17 ± 0.95 g min^−1^ (95 % CI 3.68 to 4.66) with average contribution to total energy production of 96.99 ± 2.21 % (95 % CI 95.82 to 98.15). The lowest to highest value was 90.00 to 98.85 %. Mann–Whitney *U* test revealed no difference in CHO oxidation between ATL and NATL at AnT, expressed in g min^−1^ (*U* = 207.50, *p* < 0.29) or in percent of total energy contribution (*U* = 194.00, *p* < 0.18).

## Discussion

The purpose of this study was to assess substrate oxidation during a GXT to exhaustion while determining the point at which FAT_min_ occurs in ATL and NATL. Our secondary aim was to determine if there was a correlation between VO_2_ at AnT and at the FAT_min_. Our results showed that FAT_min_ was obtained at 87.60 ± 1.60 % of VO_2max_ in ATL and at 85.25 ± 1.10 % of VO_2max_ in NATL. AnT was reached at 87.57 ± 1.30 % of VO_2max_ in ATL and 84.64 ± 1.10 % of VO_2max_ in NATL. Hetlelid et al. [13] found AnT in well-trained and recreationally trained athletes to be at 90 and 83 % of VO_2max_, respectively. Mickelson et al. [20] found AnT in elite athletes at 83 % VO_2max_. Our results coincide with previous studies confirming higher anaerobic capacities in athletes.

Pearson correlations for AnT and FAT_min_ in ATL and NATL were very high (*r* = 0.99, *p* < 0.01, 95 % CI 0.99 to 1.00 and *r* = 0.97, *p* < 0.01, 95 % CI 0.91 to 0.98), respectively. Large effect size explained 98.01 % of variance in ATL demonstrating high strength of connection. The corresponding effect size in NATL explained 94.09 % of variance.

Previous studies examining the variability of substrates utilization at high intensities did not report intensities and total fat and CHO oxidation values when RQ ≥1. Goedecke at al. [11] investigated substrate oxidation at three different intensities and reported RQ of 0.97 at last stage equaling 70 % peak power output. Van Loon et al. [29] performed measurements at different intensities, with the last stage corresponding to 72 % VO_2max_. RQ was not reported, but it was stated that fat was contributing up to 25 % to total energy production. Therefore, we estimated that last stage RQ was 0.92 ± 0.05. Stepto et al. [28] performed testing at 86 % VO_2max_ and reported RQ = 0.92. Coyle et al. [7] performed testing at 80 % VO_2max_ without reporting RQ but confirmed “high lactate threshold,” leading to an assumption that exercise intensity was under AnT. Romijn et al. [25] performed testing at 85 % VO_2max_ and reported RQ of 0.91. Hetlelid et al. [13] performed testing at 94 and 89 % of VO_2max_ and reported RQ of 0.88 and 0.95, respectively. The results of these studies suggest that, at the highest measured intensities, subjects were continuously under RQ = 1 or AnT, which may explain why fat oxidation was present.

To the best of our knowledge, there have been no studies on CHO utilization at intensities corresponding to AnT. In our study, ATL had CHO oxidation of 4.47 ± 1.24 g min^−1^ when RQ = 1.00 contributing to 97.91 ± 1.02 % of total energy production. NATL had 4.17 ± 0.95 g min^−1^ CHO utilization (RQ = 1.00) and 96.99 ± 2.21 % contribution to total energy expenditure. Rehrer et al. [24] found an average CHO oxidation to be 2.50 g min^−1^ at 70 % VO_2max_ in sedentary population, but did not report RQ. Stepto et al. [28] reported CHO oxidation of 4.95 g min^−1^ while cycling at 86 % VO_2max_ (RQ = 0.92) which is higher than in our study. Romijn et al. [25] reported CHO oxidation of 3.22 g min^−1^ in male cyclists at 85 % VO_2max_ and RQ = 0.91. Hetlelid et al. [13] reported CHO oxidation of 3.61 g min^−1^ (RQ = 0.88) at 94 % VO_2max_ and 3.79 g min^−1^ (RQ = 0.95) at 89 % VO_2max_ in well-trained and recreationally trained athletes, respectively. Our data is in agreement with these studies; nevertheless, a direct comparison with other studies is difficult due to differences in factors affecting CHO utilization (diet, pre-training meal, age, sex, weather conditions, and testing methodology).

In our study, we used a GXT treadmill protocol with 2-min stages and a constant incline of 1 %. With this type of protocol, we aimed to compensate for the lack of air resistance while running on a treadmill and to obtain more accurate and detailed sample data [6, 18]. The average duration of the exercise stage was 16 min in ATL and 12 min in NATL. Numerous authors recommend that tests for VO_2max_ should last no longer as 12 min, as prolonged tests could lead to inconsistent results [4, 23]. We noticed that athletes could not achieve VO_2max_ within 12 min due to their high endurance capacity, making short test stages more preferable. Finally, this type of protocol is highly correlative to running economy and actual VO_2max_ consumption with outdoor running. This provided additional reassurance that the protocol used is suitable for accurate measurement of VO_2max_, correlation assessment, and physiological testing of athletes in their natural environment [6, 18, 22].

A mechanism behind lipid oxidation at high intensities has not been fully elucidated. With increased intensity, there is a gradual shift from fat as a primary fuel source to CHO, until complete cessation of fat as a fuel for high-intensity exercise. This point, called FAT_min_, highly correlates with AnT in our study. It is important to note two things: (1) AnT and FAT_min_ are interchangeable because they occur at the same individual point and (2) the point at which they occur depends on several factors such as training level and activity type, sex, age, and genetics which could explain both inter-subject and inter-study differences [16].

Additional benefits for athletes and coaches can be surmised from this study. The size of the glycogen storage depends on the muscle size, with approximately 500 g locally available (additional 100 g globally available in liver), corresponding to approximately 3000 kcal of produced energy. Since there are no lipids available at intensities above AnT, CHO storage would be depleted after approximately 2 h, contributing towards the effect known as “hitting the wall,” therefore limiting not only athletes but also every subject performing at this level.

We consider as a key limitation of this study a rather small sample size which could affect our ability to estimate a causal relationship. Further studies with larger sample sizes including different types of subjects (sedentary, obese, different sports) would allow investigators to further understand how high-intensity exercise and the lipid oxidation are related. Other limitations include lack of control over the pre-test nutritional habits of the subjects which could have affected fat and CHO oxidation levels. High or low CHO diets could have some impact on total oxidation rates of the substrates making this question open for further studies.

## Conclusions

In conclusion, this is the first study to report the point at which fat utilization ends at high-intensity exercise in male athletes and non-athletes. These results confirm our hypothesis that FAT_min_ and AnT are related and highly individual and therefore can be used as a determination end point of fat oxidation and to enhance exercise performance. Our data suggest no presence of lipids and demonstrated equal CHO oxidation levels at intensities matching AnT in both test groups.

## Abbreviations

AerT: Aerobic threshold
AMP: Adenosine monophosphate
AnT: Anaerobic threshold
ATL: Athletes
CHO: Carbohydrates
FAT_max_: Maximal point of fat oxidation
FAT_max_: Point at which the fat oxidation ends
FFA: Free fatty acids
GXT: Graded exercise testing
HSL: Hormone sensitive lipase
NATL: Non-athletes
Pi: Inorganic phosphate
RQ: Respiratory quotient
SD: Standard deviation
VCO_2_: Carbon dioxide production
VO_2_: Oxygen consumption
VO2max: Maximal oxygen uptake
VT_2_: Second ventilatory threshold
